# Slowdown of photoexcited spin dynamics in the non-collinear spin-ordered phases in skyrmion host GaV_4_S_8_

**DOI:** 10.1038/s41467-022-30829-z

**Published:** 2022-06-09

**Authors:** Fumiya Sekiguchi, Kestutis Budzinauskas, Prashant Padmanabhan, Rolf B. Versteeg, Vladimir Tsurkan, István Kézsmárki, Francesco Foggetti, Sergey Artyukhin, Paul H. M. van Loosdrecht

**Affiliations:** 1 II. Physikalisches Institut, Universität zu Köln, Zülpicher Str. 77, D-50937 Köln, Germany; 2 Experimental Physics V, Center for Electronic Correlations and Magnetism, University of Augsburg, 86159 Augsburg, Germany; 3 Institute of Applied Physics, MD 2028 Chișinău, Republic of Moldova; 4 Istituto Italiano di Tecnologia, Via Morego 30, 16163 Genova, Italy; 5 Dipartimento di Fisica, Università di Genova, Via Dodecaneso, 33, 16146 Genova, Italy

**Keywords:** Magnetic properties and materials, Spintronics, Magneto-optics

## Abstract

Formation of magnetic order alters the character of spin excitations, which then affects transport properties. We investigate the photoexcited ultrafast spin dynamics in different magnetic phases in Néel-type skyrmion host GaV_4_S_8_ with time-resolved magneto-optical Kerr effect experiments. The coherent spin precession, whose amplitude is enhanced in the skyrmion-lattice phase, shows a signature of phase coexistence across the magnetic phase transitions. The incoherent spin relaxation dynamics slows down by a factor of two in the skyrmion-lattice/cycloid phases, indicating significant decrease in thermal conductivity triggered by a small change of magnetic field. The slow heat diffusion in the skyrmion-lattice/cycloid phases is attributed to the stronger magnon scattering off the domain walls formed in abundance in the skyrmion-lattice/cycloid phase. These results highlight the impact of spatial spin structure on the ultrafast heat transport in spin systems, providing a useful insight for the step toward ultrafast photocontrol of the magnets with novel spin orders.

## Abstract (web summary)

Skyrmions are a topological magnetic texture that have garnered considerable interest for various technological applications. Here, Sekiguchi et al. investigate the ultrafast optical response of GaV4S6, and find a significant reduction in the thermal conductivity in the skyrmion phase.

## Introduction

In spin systems several types of interactions can coexist, and the competition among them sometimes results in the formation of novel spin ordering, which determines the static and dynamic magnetic properties. A prominent example is the skyrmion state that originates from the presence of the symmetric and anti-symmetric spin interactions, such as ferromagnetic exchange and Dzyaloshinskii-Moriya interaction^1–4^. Magnetic skyrmions, which are particle-like spin textures with a nontrivial topology, have been investigated extensively over the past decade for their novel fundamental properties, including emergent electrodynamics leading to a topological Hall effect^5–8^ as well as their potential as an information carrier of the future memory devices^9–11^. Importantly, formation of magnetic orders modifies the character of elementary excitations, e.g., their energies, velocities, and lifetimes, that determine the dynamic properties of the spin system. This is crucial for the skyrmion device development, or spintronic information processing in general, because working principle of the memory devices, such as writing, deleting, and transfer of the skyrmions necessitate dynamic perturbations of the magnetic order. Consequently, the interest in the non-equilibrium skyrmion phenomena is currently emerging^12–23^.

It is of particular interest to drive skyrmions utilizing optical technologies. To date, several methods for the optical manipulation of magnetic order have been established^24–28^, and have recently been extended to skyrmion hosts^14,17–23^. It is demonstrated that coherent spin precession can be triggered in skyrmion-host materials by ultrafast optical pulses^14,23^. The excitation efficiency of coherent magnons depends on the geometrical relation between the photoexcitation and spin ordering, i.e., the coupling strength of the photoinduced magnetic perturbation and the oscillating moment of the specific magnon mode. Therefore, detailed investigation of the coherent magnons depending on the nature of skyrmion ordering is highly demanded for the coherent photocontrol of skyrmions.

Furthermore, laser-based time-resolved studies also shed light on the thermal properties of the spin system through the observation of incoherent relaxation dynamics^24,29,30^. The ability to control thermal currents is of paramount importance, especially for realizing prospective high-density spintronic information processing units. Investigations into means to control heat flow in magnetic systems gave rise to the field of spin caloritronics^31^. For example, a heat valve effect has been demonstrated in sandwich nanostructures with ferromagnetic and non-magnetic layers^32^. As mentioned earlier, the nature of magnon modes is modified by the spin ordering, that can affect the thermal transport properties. However, the influence of the complex spin order on thermal conductivity remains hitherto largely unexplored.

In this study, we investigate dynamic behavior of the spin system with different magnetic orders of the bulk Néel-type skyrmion host material GaV_4_S_8_, by performing time-resolved magneto-optical Kerr effect (trMOKE) experiment across the magnetic phase diagram, i.e., the ferromagnetic (FM) phase, skyrmion-lattice (SkL) phase and cycloid (Cyc) phase. It is previously reported that the coherent spin precession in GaV_4_S_8_ can be optically driven by the sudden suppression of magnetic anisotropy^23^. Here, we show that the amplitude of this coherent spin motion is enhanced in the SkL phase owing to the efficient coupling between the photoinduced magnetic perturbation and the skyrmion breathing mode. Furthermore, the incoherent remagnetization dynamics becomes slow in the SkL and Cyc phases, suggesting a strong suppression of spin thermal conductivity. By comparing with atomistic spin dynamics simulations, this behavior is attributed to strongly enhanced magnon scattering due to the multi-domain magnetic structure, consistent with the signature of phase coexistence observed in the coherent spin precession experiments.

## Results

### Magnetic properties and phase diagram of GaV_4_S_8_

**Fig. 1:**
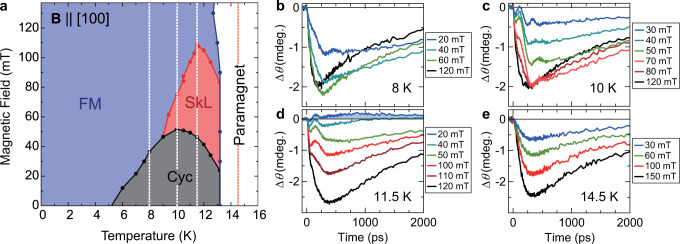
Photoinduced Δ*θ* dynamics in the different magnetic phases of GaV_4_S_8_. **a** Magnetic phase diagram of GaV_4_S_8_. Vertical dashed lines indicate the temperatures where trMOKE measurements were performed. **b**–**e** Δ*θ* traces under different external magnetic fields at 8 K, 10 K, 11.5 K, and 14.5 K, respectively.

GaV_4_S_8_ is a multiferroic semiconductor from the lacunar spinel family, with a non-chiral polar crystal structure. The magnetic phase diagram of GaV_4_S_8_ is shown in Fig. 1a; below the Curie temperature of *T*_C_ ~13 K, the Cyc phase and SkL phase appear in addition to the FM phase^33^. The SkL phase in GaV_4_S_8_ extends over a wide region in the phase diagram, which facilitate the study of the dynamical properties of the skyrmions. Below ~44 K, a cooperative Jahn-Teller distortion along one of the cubic body diagonal axes drives a transition from a cubic to polar rhombohedral structure, resulting in the formation of a submicron-thick lamella-type multi-domain structure^33,34^. We note that in the sister compound, GaV_4_Se_8_, the polar structural domain walls were recently reported to host twisted magnetic states different from those in the interior of the domains^35^. In the rhombohedral phase of GaV_4_S_8_, uniaxial anisotropy develops with the magnetic easy axis parallel to the polar rhombohedral axes, being either of the cubic <111> axes^33,34,36^. The temperature dependence of this anisotropy has been investigated by electron spin resonance (ESR) spectroscopy below the magnetic ordering temperature, *T*_C_ ~13 K^36^. It was also demonstrated that the uniaxial anisotropy has a strong impact on the stability range of the SkL phase in lacunar spinels^33,34,37,38^, and specific to GaV_4_S_8_ it confines the orientation of the skyrmion tubes along the easy axis^33,37^.

In the trMOKE experiments, the external magnetic field is applied normal to the (100) crystal surface so that all of the four possible structural domains host the same magnetic state, since the magnetic field spans the same angle with the four <111> directions being the magnetic easy axis of the four possible domains. Hence, these four domains contribute equally to the trMOKE signal. The experiments were performed with a Yb:KGW regenerative amplified laser as main light source, which provides higher pulse energy with lower repetition rate (100 kHz) compared to the setup in our previous work^23^. This enables stronger photoexcitation while minimizing heat accumulation in the sample. The experiments have been performed in the regime where the response is linear in pump fluence (~30 *μ*J/cm^2^ on the sample surface). For the pump and probe, infrared pulses (800 nm, 40 fs) and visible pulses (515 nm, 270 fs) were used, respectively. The pump pulse was linearly polarized, with the photon energy (1.55 eV) well above the GaV_4_S_8_ band gap of ~0.35 eV^39,40^.

### Coherent spin precession

Figures 1b–e show the photoinduced transient change of the Kerr rotation angle of the probe pulse, Δ*θ*, for the magnetic fields ranging from 20–150 mT and four fixed temperatures as indicated by the dashed vertical lines in the phase diagram Fig. 1a. The observed Δ*θ* traces show the presence of several processes characterized by their timescales. First, immediately after photoexcitation Δ*θ* shows a marked fast (<40 ps) decrease. This behavior is observed at all magnetic fields and all temperatures below *T*_C_ as can be seen in Figs. 1b-1d. This initial fast demagnetization stems from the photoexcited carrier relaxation process, where magnons are created either through direct electron-magnon scattering or by the phonons emitted during non-radiative carrier relaxation process^41^. After this initial process, Δ*θ* shows a relatively slow demagnetization, and an even slower subsequent remagnetization. The demagnetization and remagnetization processes also occur in the paramagnetic phase slightly above *T*_C_. Because GaV_4_S_8_ is a semiconductor, the demagnetization process is completed in a relatively slow timescale of ~200–400 ps, as widely observed in semiconductor magnets^24^. In general, in semiconductors this slow demagnetization dynamics is understood in terms of spin-lattice thermalization, which takes place after the rapid heating of the lattice system due to an efficient energy transfer from the electron system to the lattice system^24^. An interesting observation is that at 11.5 K and at a low magnetic field Δ*θ* changes sign and takes a positive value, indicating that magnetization is enhanced by the photoexcitation. The origin of this lies in the peak structure of magnetic susceptibility around the phase boundary between the Cyc and paramagnetic phases^23,42^. In addition to these incoherent spin dynamics, a clear oscillatory structure appears in the magnetically ordered phase with a typical period of 100–300 ps, i.e., in the few GHz range. The excitation mechanism of this coherent collective spin motion has been assigned to the ultrafast photoinduced modulation of the magnetocrystalline anisotropy of GaV_4_S_8_^23^.

**Fig. 2:**
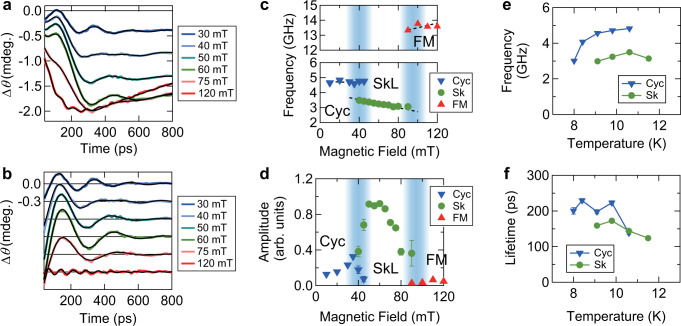
Coherent spin precession in the FM, SkL, and Cyc phases. **a** Δ*θ* traces in the first 800 ps shown with fit curves (black solid lines) at 10 K. **b** Coherent oscillatory component of the data shown in **a**. **c**, **d** Magnetic-field dependence of the frequency and amplitude of the coherent oscillation. The blue-shaded areas are guides to the eye indicating where the magnetic phase transition occurs. **e**, **f** Temperature dependence of the frequency and lifetime of the coherent oscillation, with the magnetic field fixed at 30 mT (60 mT) for the Cyc (SkL) phase. Error bars are the standard deviation of the fitting parameters.

The comprehensive set of the Δ*θ* traces clearly shows that the nature of the coherent and incoherent spin dynamics changes character depending on the location in the magnetic phase diagram. We first discuss the coherent oscillatory response in the different magnetic phases. To do this, we fit the data using a damped cosine oscillation, superimposed on a bi-exponential function that accounts for the incoherent demagnetization and remagnetization dynamics. We disregard the dynamics on the earliest timescale (up to ~40 ps) originating from ultrafast carrier relaxation processes [see supplementary information]. Figure 2a shows data taken at *T* = 10 K for various fields in the Cyc, SkL, and FM phases, along with the phenomenological model fits to the data (black lines). To more clearly show the coherent oscillatory component, we replot the data in Fig. 2b after subtracting the incoherent component. The magnetic-field dependence of the oscillation frequency is shown in Fig. 2c. At low magnetic fields in the Cyc phase, the frequency of the oscillation lies around ~4.7 GHz, which drops to ~3.5 GHz near the phase transition to the SkL phase. These frequencies, which have also been observed in the ESR experiments^43^, correspond to the low-frequency collective mode in the Cyc phase, and to the skyrmion breathing mode in the SkL phase, respectively. Further increase of the magnetic field finally causes the transition to the FM phase around 100 mT, which is manifested in a substantially higher-frequency (~13.5 GHz) coherent oscillation corresponding to the ferromagnetic resonance in the FM phase^44^.

A distinct feature of the skyrmion phase is observed in the amplitude of the coherent oscillation. Figure 2d shows the magnetic-field dependence of the amplitude, where a drastic increase is observed inside the SkL phase. This enhanced amplitude in the SkL phase can be attributed to the efficient excitation of the breathing (BR) mode of the skyrmions. Due to the magnetocrystalline anisotropy, skyrmion tubes in GaV_4_S_8_ keep co-aligned with the easy axis and do not follow the orientation of the magnetic field^33^. In addition, anisotropy also affects the skyrmion core size. In the SkL phase, the spin system acquires a collective motion of the skyrmion BR mode, accompanied by the oscillating magnetization components along the skyrmion tube direction^45,46^. On the other hand, the coherent spin precession is triggered by the photoinduced quench of the magnetocrystalline anisotropy^23^, as well as by the change of the *g*-tensor as the Jahn–Teller distortion is diminished by the photoexcitation. Therefore, the skyrmion BR mode strongly couples to the photoinduced anisotropy quench, resulting in the large amplitude of the coherent oscillation, whereas in other phases such an efficient excitation does not occur. This observation demonstrates that the skyrmion-specific coherent dynamics is not only distinguished by the mode frequency but also by the high photoexcitation efficiency.

In Fig. 2d, with increasing magnetic field, the amplitude strongly but continuously increases when the system undergoes the Cyc to SkL phase transition. It saturates with a maximum value around 60 mT in the SkL phase, then gradually decreases as the system approaches the FM phase. This behavior reflects phase coexistence near the magnetic phase boundaries, i.e., when entering the SkL phase, skyrmions are created in the form of islands inside the Cyc (FM) phase^2,47^. As the system approaches the center of the SkL phase, the fraction of the skyrmion phase increases, in other words the system becomes more “pure” SkL phase, which can be observed in the enhanced and eventually saturated amplitude of the oscillation. The signature of the phase coexistence can also be visually identified in Δ*θ* traces. In Figs. 2a and 2b, the trace at 40 mT shows a small amplitude second oscillation, which is expected to form a dip structure around 300–500 ps as seen in the traces at 30 mT and 50 mT. The trace at 40 mT, which is near the phase boundary between the Cyc and SkL, is best described by the presence of two coherent oscillations corresponding to those adjacent phases. This time-domain observation of the phase coexistence is in line with the low-frequency AC susceptibility measurement where an extremely slow spin dynamics appears in the vicinity of the phase boundaries^48^.

The lifetime of the coherent oscillation at *T* = 10 K is around 200 ps, without significant variations for different phases [see also supplementary information]. On the other hand, Δ*θ* traces shown in Fig. 1d show that the oscillation becomes heavily damped at *T* = 11.5 K. The temperature dependence of the lifetimes at fixed magnetic field is plotted in Fig. 2 f, which shows the lifetime becoming shorter upon increasing temperature, both for the Cyc and SkL phases. Finally, we note that, while the frequency of the coherent oscillation in the SkL phase is fairly temperature independent, the cycloid oscillation mode shows a softening toward lower temperatures (see Fig. 2e). This may seem counterintuitive from the naive expectation that the frequency should scale with the magnetization. The Cyc phase, however, has two collective modes (±*Q* modes)^49,50^ which are split into a lower-frequency (lf) and higher-frequency (hf) mode, of which only the lowest mode is efficiently excited in this experiment^23^. The splitting of these modes is due to dipole interaction and/or magnetic anisotropy^49,50^, which both increase toward lower temperature and hence push the lf-mode to the lower frequency at lower temperature.

### Remagnetization dynamics

**Fig. 3:**
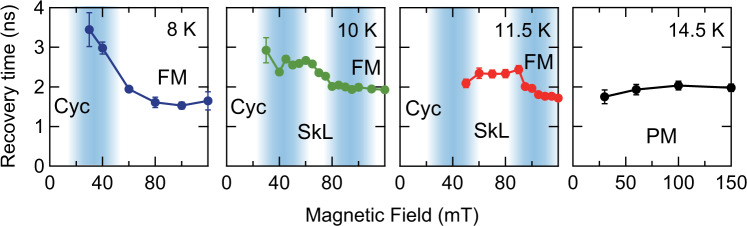
Change of the remagnetization across the magnetic phase transition. Time constant of the remagnetization process at 8 K, 10 K, 11.5 K, and 14.5 K are plotted. The blue-shaded areas are guides to the eye indicating the magnetic phase transition. Error bars are the standard deviation of the fitting parameters.

The coherent spin precession observed above shows a signature of phase coexistence across the magnetic phase transitions in GaV_4_S_8_. This observation indicates the formation of magnetic domain walls in the course of phase transitions, which will also exist inside the SkL/Cyc phases^2,47^. In connection with this, the incoherent remagnetization dynamics shows an interesting behavior. A quick inspection of the Δ*θ* traces in Fig. 1 reveals that the remagnetization dynamics is slower at lower magnetic fields, compared to that in the higher-field FM phase. For a quantitative discussion on this later timescale, we extract the time constant of the remagnetization using an exponential fit to the Δ*θ* traces after 1 ns. Figure 3 shows the magnetic-field dependence of the remagnetization time at three different temperatures. Here the data at low magnetic fields, where the fits become less reliable due to the long time constants compared to the experimental time window, are excluded. At 14.5 K in the paramagnetic (PM) phase, no characteristic dependence on the magnetic field is observed. However, below *T*_C_ the remagnetization time slows down across the phase transition from the FM phase to the SkL phase (11.5 K and 10 K) and the Cyc phase (8 K). The increase of the remagnetization time reaches a factor of 1.5–2 depending on the temperature, which is remarkable considering that it is triggered by a small change of magnetic field in a single material.

As described earlier, the spin system is heated up through the spin-lattice coupling in 200–400 ps, and afterwards the recovery of the magnetization occurs due to the heat diffusion away from the excited region. Indeed, the remagnetization time of a few ns is consistent with the timescale estimated from the heat diffusion, assuming 1-dimensional diffusion of a single thermal substance. This assumption is reasonable considering the large photoexcitation spot size (~600 μm), thin penetration depth of the photoexcitation (<1 μm)^40^ and small diffusion length less than few μm on the ps-ns timescale estimated using a typical value of thermal conductivity of 1–100 W/mK^51^. Therefore, we can attribute the slowing down of the remagnetization dynamics in the SkL/Cyc phases to a suppression of the thermal conductivity.

Thermal conductivity is determined by the energy, velocity, and mean free path of the heat-carrying quasiparticles. While the magnetic phase is spatially uniform in the FM phase, dislocations are possible in the skyrmion lattice of the SkL phase, and domains with different orientations of wave vectors form upon the transition to the Cyc phase. Indeed, in the SkL phase, the spin correlation length for the skyrmion-lattice in-plane direction is much shorter than that for the skyrmion-core direction, estimated from the width of the Bragg peaks of small-angle neutron scattering measurements^33^. This confirms the existence of magnetic multi-domain structure in GaV_4_S_8_. These types of magnetic discontinuities lead to an enhanced magnon scattering and thereby, reduce the magnon mean free path. As a result, the magnetic heat transport is reduced by the formation of magnetic domain boundaries, as for instance has been reported for the helimagnetic insulator Cu_2_OSeO_3_^51^.

In addition to the reduced mean free path, the magnon velocity also depends on the magnetic phase. Because the SkL and Cyc phase in GaV_4_S_8_ have a long spin periodicity (the SkL (Cyc) periodicity is ~22 nm (~18 nm)), the Brillouin Zone of the magnon dispersion folds into a small momentum (*q*) space. This results in relatively flat magnon branches^52,53^, rendering the effective magnon velocity slow. This seems qualitatively consistent with our experimental observations. However, such a modulation of magnon dispersion is prominent in the small wave vectors corresponding to the long pitch of the skyrmion-lattice or spin cycloid, and in the lower energy scale of ~ μeV. Indeed, the eigenmode of spin oscillations in the SkL or Cyc phase appears in the ~GHz frequency with the zero magnon velocities. The contribution of the magnons with such small velocities to the heat transport is limited, and hence the thermal transport is considered not much affected by the change of magnon velocity across the phase transition to the SkL and Cyc. On the other hand, the formation of magnetic domain walls reducing the magnon mean free path induces the broadening in the magnon dispersion at finite wave vector in the thermal energy range of ~ meV (at 10 K), as shown in detail later.

Moreover, there are other magnetic-phase-dependent parameters affecting the remagnetization dynamics, namely the spin heat capacity and the spin-lattice coupling. Here, the spin heat capacity is reported to be smaller in the SkL and Cyc phase compared to FM phase^42^, which by itself does not explain the slower recovery dynamics in the SkL/Cyc phase. The spin-lattice coupling can affect the thermal relaxation dynamics through the phonon-magnon scattering processes^54^. The efficiency of phonon-magnon scattering processes depends on the character of the magnon modes, which changes across the magnetic phase transition^55^. However, the length scale of the spin periodicity in the SkL and Cyc phase are around 20 nm, which is much longer than the unit cell scale. This results in the modulation of the magnon modes at very low frequency of ~GHz with small wave vector, as mentioned earlier. The impact of such a spin modulation with *q *~ 0 on the phonon velocity is considered to be limited. In other words, since the spin texture in all the considered phases is essentially ferromagnetic on a unit cell length scale, the magnon modes in the SkL and Cyc phases are not expected to have very different magnon-phonon scattering rates, and will remain similar to the rate in the FM phase. It is also possible that the magneto-elastic coupling induces local structural distortions at the magnetic domain boundaries, which enhances the phonon scattering. However, the contribution of this magnetization-dependent process to the total phonon scattering rate is expected to be limited in GaV_4_S_8_. Due to the modest magnitude of magnetostriction in GaV_4_S_8_, magnetization-induced structural distortions are tiny. In contrast, the Jahn–Teller distortion associated with the cubic to rhombohedral structural transition at *T*_JT_ ~44 K are more drastic. This is confirmed by the fact that the polarization change across the magnetic phase transition is only ~0.1 % compared to that of the structural transition across *T*_JT_^42^. Therefore, structural domain walls associated with the Jahn–Teller distortion are expected to have a dominant contribution to the phonon scattering and hence on the phonon mean free path. Since the Jahn–Teller distortion is not sensitive to the magnetization, it is expected that the phonon scattering rate hardly changes upon crossing the magnetic phase transitions. On the other hand, the magnetic domain walls significantly affect magnons as discussed in the following.

**Fig. 4:**
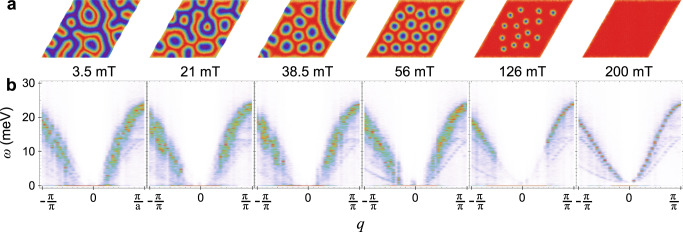
Simulated spin texture and magnon spectra. **a** Representative spin textures and **b** magnon spectral functions, obtained from atomistic spin dynamics simulations of GaV_4_S_8_ at *T* = 12 K and magnetic field magnitudes, as indicated at the top of each panel.

In order to gain a better insight into the magnon-mediated thermal conductivity in various magnetic phases of GaV_4_S_8_, we simulated a microscopic spin model of GaV_4_S_8_. The dominant interactions in GaV_4_S_8_ are ferromagnetic intralayer and interlayer Heisenberg exchange coupling^56^. At the temperature around 12 K and high magnetic fields the FM state is stabilized. With decreasing magnetic field GaV_4_S_8_ enters the SkL state, which transforms into the Cyc state at even lower fields, as seen in Fig. 4. The details of the simulations leading to these results are described in the Methods Section.

**Fig. 5:**
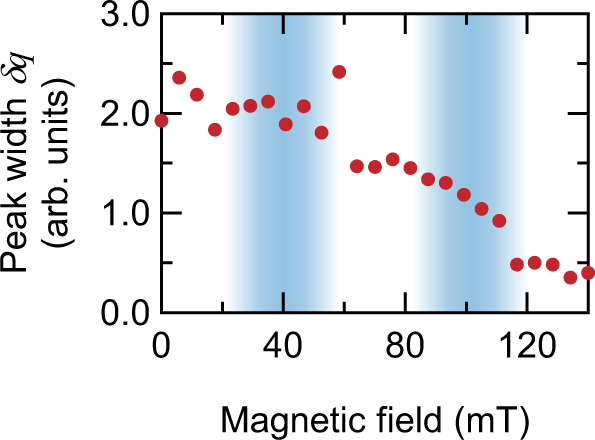
Magnon peak width extracted from simulated spectral functions. A Gaussian fit of magnon peaks is performed on the cuts of the magnon spectral function S(*q*, $\omega$ = 8 meV) and the magnon peak width $\delta q$ is determined. Plotted is the magnetic field dependence. The blue-shaded areas are guides to the eye indicating the magnetic phase transition.

The representative configurations at *T* = 12 K are shown in Fig. 4a. Whereas the spin configuration is spatially uniform in the FM state, dislocations in the SkL phase and domain walls in the Cyc are formed because the spiral states with different wave vector orientations are degenerate^2,23,33,47^. Such a magnetic multi-domain structure results in the incoherent magnon excitations, which can be identified by the magnon linewidth increasing towards the non-collinear spin states. Figure 4b shows the simulated magnon spectral function (for more detail, see Methods), and the evolution of the magnon broadening across the phase transitions is shown in Fig. 5. The reduction of the spin correlation length in the SkL and Cyc phase considerably lowers the magnon mean free path, encoded by the inverse width of the magnon lines in the spectral function.

The magnon thermal conductivity is given by $\kappa ={\sum }_{i}{c}_{i}{v}_{i}{\rho }_{i}$, where ${c}_{i}$ is the specific heat of mode $i$, ${v}_{i}$ is its velocity and ${\rho }_{i}$ is the mean free path. The latter can be estimated via the magnon spectral broadening as ${\rho }_{i}=2\pi /\delta q$, with the $\delta q$ is the line broadening. As discussed earlier, ${c}_{i}$ and ${v}_{i}$ do not play a decisive role in this experiment, and hence $\kappa$ is inversely proportional to $\delta q$. The heat transfer equation reads $\frac{c\partial T}{\partial t}=\kappa \,\Delta T$, so that the relaxation time scale is $\tau \sim c\,/\,\kappa$. Therefore, the relaxation time, inversely proportional to the thermal conductivity, is expected to change along the same trend as the magnon linewidth $\delta q$. Indeed, the experimental data show significant increase of the relaxation time in the SkL and Cyc phases. Larger errors of the remagnetization time constant at low fields in the experiment make a quantitative comparison difficult in this region, but the overall trend of decreasing thermal conductivity in the non-collinear SkL and Cyc phase compared to the FM phase is robust. Such domain-wall driven thermal conductivity decrease is analogous to that observed in ferroelectric Pb(Zr_0.3_Ti_0.7_)O_3_, where a decrease of phonon thermal conductivity of 11% has been achieved by manipulating the domain structure by means of external electric field^57^.

To summarize, we investigated the ultrafast spin dynamics in different magnetic phases in GaV_4_S_8_ by using time-resolved MOKE experiments. The formation of skyrmions manifests itself not only in the frequency change of the collective mode, but also in the enhanced excitation efficiency of the coherent spin oscillation. The field dependence of the excitation efficiency of coherent spin precession modes shows a fingerprint of phase coexistence of, for instance, the SkL and Cyc phases, suggesting the formation of magnetic domain walls. In line with this observation, the formation of the skyrmions and spin cycloid induces slow magnetization recovery, which can be attributed to the enhanced magnon scattering off the magnetic domain walls. This can be regarded as a giant heat valve effect, realized here in a single material and triggered by a small magnetic field change necessary for the transition from the FM to SkL/Cyc state. This result unlocks a new door to thermal management in future spintronic devices. From a broader perspective, our study demonstrates that the change of spin ordering triggers a dramatic change of dynamic behaviors, motivating further research on materials with novel spin correlations.

## Methods

### Sample preparation and characterization of the magnetic properties

GaV_4_S_8_ single crystals have been grown by the chemical transport reactions method in a way similar to Ref. ^55^. As starting material for growth, the preliminary synthesized polycrystalline powder was used. The polycrystals were prepared by solid state reactions using high-purity elements: Ga (99.9999%), V (99.5%) and S (99.999%). The iodine was utilized as the transport agent. The crystal growth was performed at temperatures between 800 and 850 °C. Perfect truncated octahedron-like samples with shiny faces and dimension up to 5 mm were obtained after 2 month of transport.

Magnetic properties were measured by a SQUID magnetometer (MPMS 5, Quantum Design) in the temperature range 1.8–400 K in magnetic fields up to 5 T. Mapping of the magnetic phases shown in phase diagram, Fig. 1a of the paper, was done using the data on derivatives d*M*/d*H*, taking the singularity points as the phase boundaries between the cycloidal (Cyc), skyrmion (SkL), and ferromagnetic (FM) phases^33^.

### Time-resolved magneto-optical Kerr effect experiment

The experiments were performed with a Yb:KGW regenerative amplified laser as main light source (Pharos) with the repetition rate of 100 kHz. Infrared output of a non-collinear optical parametric amplifier (800 nm, 40 fs) and the second harmonic generation of the fundamental light (515 nm, 270 fs) were used for the pump and probe pulse, respectively. The spot size of the focused pump pulses on the sample was ~600 *μ*m in diameter, larger than that of the probe pulse ~250 *μ*m. The pump pulse was linearly polarized, whose photon energy (1.55 eV) lies well above the GaV_4_S_8_ band gap of ~0.35 eV^39,40^. The pump-induced transient change of the Kerr rotation angle of the probe pulse, Δ*θ*, was measured. The sample temperature and external magnetic field were controlled with a superconducting-magnetic cryostat.

### Modelling of the spin texture and calculation of the magnon spectra

To reproduce the Cyc-SkL-FM sequence of magnetic phases, we used UppASD code^58^ to perform Monte-Carlo (MC) simulations using 72*72*1 supercells, and 100,000 Metropolis MC steps for each combination of temperature and magnetic field. The parameters of the magnetic Hamiltonian were adopted from Ref. ^56^. Particularly, we used the set of constants, obtained using superexchange theory. In order to compute magnon spectra, the MC calculations were followed by spin dynamics simulations using the UppASD program^58^. The magnon spectral function, evaluated numerically from the time-dependent correlation function

1 $${C}^{j}\left(r-{r}^{{\prime} },t\right)=\big \langle {{{{{{\boldsymbol{m}}}}}}}_{r}^{j}\left(t\right){{{{{{\boldsymbol{m}}}}}}}_{{r}^{{\prime} }}^{j}\left(0\right)\big\rangle - \big \langle {{{{{{\boldsymbol{m}}}}}}}_{r}^{j}\left(t\right)\big\rangle \big\langle {{{{{{\boldsymbol{m}}}}}}}_{{r}^{{\prime}}}^{j}\left(0\right)\big\rangle$$

of the Cartesian component *j* of the magnetization ${{{{{{\boldsymbol{m}}}}}}}_{r}^{j}\left(t\right)$ at the position **r** and time *t*. The magnetization ${{{{{{\boldsymbol{m}}}}}}}_{r}^{j}\left(t\right)$ was obtained by integration of the Landau–Lifshitz equation^59^. The Gilbert damping was set to 10^−3^. 20,000 timesteps of 10 fs were used for the integration. The Fourier transform of the correlation function gives the dynamical structure factor,

2 $${S}^{j}({{{{{\boldsymbol{q}}}}}},\omega )=\frac{1}{\sqrt{2\pi }N}\mathop{\sum}\limits_{{{{{{\boldsymbol{r}}}}}},{{{{{\boldsymbol{r}}}}^{\prime}}} }{e}^{-i{{{{{\boldsymbol{q}}}}}}\cdot ({{{{{\boldsymbol{r}}}}}}-{{{{{\boldsymbol{r}}}}^{\prime}}} )}\int_{-\infty }^{+\infty }{e}^{i\omega t}{C}^{j}({{{{{\boldsymbol{r}}}}}}-{{{{{\boldsymbol{r}}}}^{\prime}}} ,t)dt$$

The magnon linewidth was then obtained by fitting the peaks of the spectral function with Gaussians. The thermal conductivity was estimated from the linewidth as explained in the main text.

## Supplementary information

Supplementary Information

## Data Availability

The data that support the findings of this study are available from the authors upon reasonable request.

## Code availability

The codes that support the calculations of this study are available from the authors upon reasonable request.
