# Supplementary material for: Slowdown of photoexcited spin dynamics in the non-collinear spin-ordered phases in skyrmion host GaV4S8
Source: Nat Commun. 2022 Jun 9;13:3212. doi: 10.1038/s41467-022-30829-z (PMC9184521; doi:10.1038/s41467-022-30829-z)
Supplement: Supplementary file 1 — Supplementary Information [file 41467_2022_30829_MOESM1_ESM.pdf]

Supplementary Information for

Slowdown of photoexcited spin dynamics in the non-collinear spin-ordered phases in skyrmion host GaV_4S_8

Fumiya Sekiguchi[1], Kestutis Budzinauskas[1], Prashant Padmanabhan[1], Rolf B. Versteeg[1], Vladimir Tsurkan[2,3], István Kézsmárki[2], Francesco Foggetti[4,5], Sergey Artyukhin[4] and Paul H. M. van Loosdrecht[1]

[1]*II. Physikalisches Institut, Universität zu Köln, Zülpicher Str. 77, D-50937 Köln, Germany*
[2]*Experimental Physics V, Center for Electronic Correlations and Magnetism, University of Augsburg, 86159 Augsburg, Germany*
[3]*Institute of Applied Physics, MD 2028, Chișinău, R. Moldova*
[4]*Istituto Italiano di Tecnologia, Via Morego 30, 16163 Genova, Italy*
[5]*Dipartimento di Fisica, Università di Genova, Via Dodecaneso, 33, 16146 Genova, Italy*

Fit function for the analysis of the coherent spin oscillation

In this experiment, the coherent spin precession is triggered by the photoinduced change of the magnetocrystalline anisotropy[1,2]. In GaV_4S_8, the strength of the uniaxial magnetic anisotropy H_{ani}, with the easy axis parallel to the [111] polar rhombohedral axis, depends on the temperature[3]. Therefore, the photoinduced heating of the sample weakens the strength of H_{ani}. With the external magnetic field H_{ex} applied in the direction of [100] axis, such a suppression of the H_{ani} results in the change of the effective magnetic field H_{eff} acting on the spin system, as schematically shown in Fig. S1(a). This process occurs in a timescale faster than the collective modes of the spin system, allowing to trigger the coherent collective spin precession. On the other hand, at a longer timescale the strength of H_{ani} recovers due to the heat diffusion away from the photoexcited region.

For the quantitative analysis of the coherent spin dynamics, we used a phenomenological function to fit the time-resolved magneto-optical Kerr effect (trMOKE) traces. To find an appropriate fit function, we first simulate the spin motion triggered by the ultrafast modulation of H_{eff} explained above, by using the Landau-Lifshitz-Gilbert (LLG) equation. In the simulation, motion of a single spin (i.e., without spin-spin interaction) is calculated, with the initial state where the spin is aligned along H_{eff}. The magnetization component along the [100] axis (or, in other words, in the direction of applied field H_{ex}) is defined as m_z. The strength of H_{ani} is modulated by 1 % with a decay time of 40 ps and recovery time of 2 ns, as shown in Fig. S1(b). The time constants were set so that they correspond to the ultrafast demagnetization and slower magnetization recovery dynamics observed in the trMOKE traces. However, changes of the time constants nor the amplitude of the suppression of H_{ani} do not affect the spin dynamics qualitatively, and thus the consideration of the model function for the fit does not depend on the particular choice of the parameters used in the calculation.

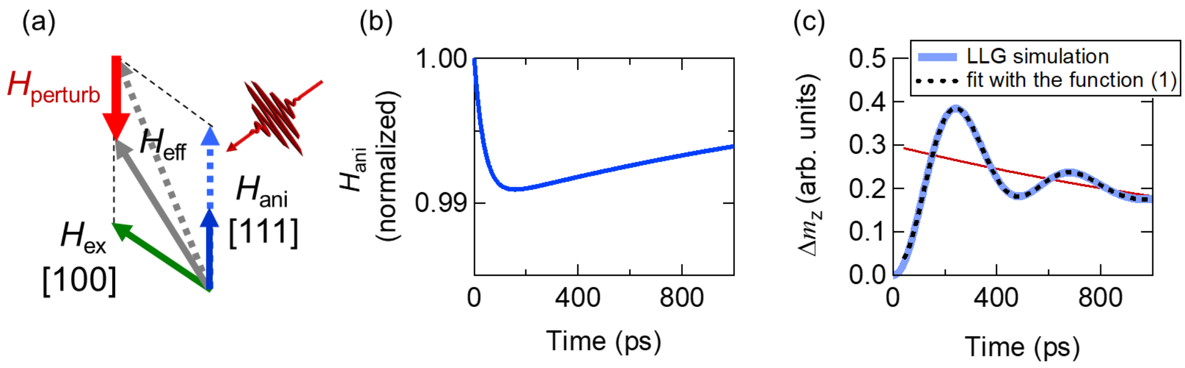

Fig. S1. Coherent magnetization dynamics induced by the photoexcitations. (a) Schematic picture of the photoinduced modulation of the effective magnetic field acting on the spin system. The magnetic anisotropy is quenched by the photoexcitation. (b) Time evolution of H_{ani} (suppression and recovery) used to calculate the magnetization dynamics. (c) Blue solid line: m_z dynamics calculated by the LLG equation. Black dashed line: fit curve with the phenomenological model function (1). Red solid line: the component representing the oscillation center in the function (1).

For the analysis of the coherent spin precession, we disregard the earliest timescale before 40 ps where the photoexcited carrier relaxation process is taking place. In this time window, H_{eff} is already shifted to a new direction around which the spin starts to rotate. However, the shifted H_{eff} slowly recover to the original direction. This behavior results in the Δm_z time trace of the damped cosine oscillation with the decaying oscillation center.

Therefore, for the coherent magnetization dynamics we used a phenomenological function described as

$$f(t) = A\left[\exp\left(-(t-t_c)/\tau_2\right) - \cos\left(\omega(t-t_c)\right)\exp\left(-(t-t_c)/\tau_3\right)\right] \qquad (1)$$

The first term in eq. (1) represent the oscillation center, which decays in a relatively long time scale τ_2. The second term represent the oscillatory component, with the oscillation ω and the damping $1/\tau_3$. The time origin is slightly shifted with the fit parameter t_c to account for the fact that the demagnetization dynamics taking place before $t = 40$ ps is excluded from the analysis. The function (1) reproduces the time trace Δm_z obtained from the LLG equation, as shown in Fig. S1(c). We note that Δm_z is positive because m_z increases by the suppression of H_{ani}, as can be understood by observing the image of Fig. S1(a).

In reality, the incoherent demagnetization dynamics also takes place and contributes to trMOKE signal. Therefore, to fit the experimental data we used a function described as

$$\Delta\theta(t) = A[\exp(-(t-t_i)/\tau_1)][1-\exp(-(t-t_i)/\tau_2)] \\ -B[\exp(-(t-t_c)/\tau_2)-\cos(\omega(t-t_c))\exp(-(t-t_c)/\tau_3)] \quad (2)$$

The first and second term corresponds to the incoherent and coherent magnetization dynamics, respectively. Those two terms have the opposite sign because the incoherent thermal dynamics induces demagnetization whereas the coherent dynamics results in the increase of m_z as shown before. The two terms share the common decay time τ_2 which represent the recovery dynamics due to the heat diffusion.

Magnetic-field dependence of the coherent oscillation lifetime

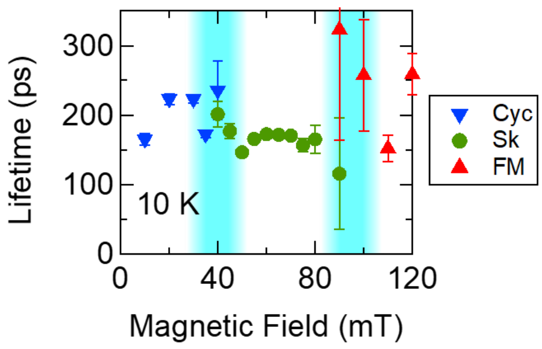

Figure S2. Magnetic-field dependence of the lifetime of coherent oscillation at 10 K.

Transient reflectivity

For comparison to the trMOKE measurement, the transient reflectivity (TR) was also measured under the same experimental conditions. As described in detail in the following, the TR data in GaV_4S_8 mainly reflect the photoinduced free carrier dynamics as widely observed in semiconductors.

TR traces at 10 K and at different magnetic fields are shown in Fig. S3(a). These data show that the TR dynamics hardly changes across the magnetic phase transitions. Figure S3(b) shows that the TR trace are composed of double-exponential function (fast rise and relatively slow decay) and slow sine-type oscillation. The latter probably comes from surface acoustic oscillation excited by the pump-pulse.

The former part of the TR response can be attributed to the photoinduced dynamics of the electronic system. It is an important fact that GaV_4S_8 is a semiconductor with a relatively narrow gap of 0.35 eV. In semiconductors, photoexcitations above the gap energy induce carriers, which significantly affects the optical properties[4]. It is not a trivial question how the photoexcited carriers affect the reflectivity under our specific experimental conditions because a TR signal can change its amplitude and even its sign depending on the electronic structure of the semiconductor and the probe wavelength. Still, the density of photoexcited carriers is roughly estimated to be around 5×10^{17} cm^{-3} from the photoexcitation fluence, which is, though fairly low, high enough to modulate the optical properties in semiconductors. In general, photoexcited carriers first emit their excess energy (i.e., the difference between the photoexcitation energy and the band gap energy) and approach equilibration with the lattice and/or spin system, and subsequently decay through radiative and/or non-radiative recombination processes. These dynamics result in the double-exponential like time traces, which is indeed observed in Fig. S3(a).

It is interesting that the TR dynamics are not noticeably affected by the magnetic phase transitions, whereas the trMOKE dynamics changes dramatically. This reflects the fact that TR and trMOKE monitor dynamics of different subsystems. The TR signal monitor the diagonal parts of response function which is at best only weakly dependent of the magnetization, whereas trMOKE monitors time evolution of the off-diagonal parts, which explicitly reflect the magnetization, and hence its dynamics in our experiments.

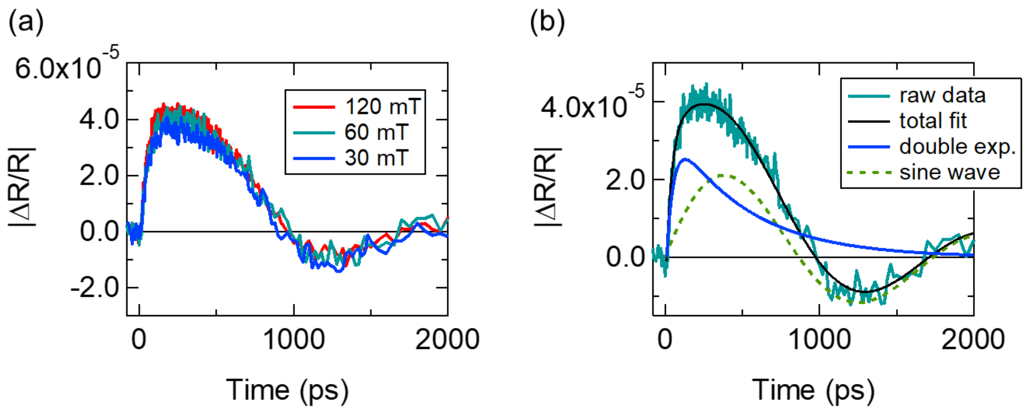

Figure S3. Transient reflectivity data. (a) TR traces at 10 K and at different magnetic fields. (b) Decomposition of the TR trace at 60 mT into double-exponential function (fast rise and relatively slow decay) and slow sine-type oscillation.

References

1. Kirilyuk, A., Kimel, A. V. & Rasing, T. Ultrafast optical manipulation of magnetic order. *Rev. Mod. Phys.* **82**, 2731–2784 (2010).

2. Padmanabhan, P. *et al.* Optically Driven Collective Spin Excitations and Magnetization Dynamics in the Néel-type Skyrmion Host GaV_4S_8. *Phys. Rev. Lett.* **122**, 107203 (2019).

3. Ehlers, D. *et al.* Exchange anisotropy in the skyrmion host GaV_4S_8. *J. Phys. Condens. Matter* **29**, 65803 (2017).

4. Shah, J. Ultrafast Spectroscopy of Semiconductors and Semiconductor Nanostructures (Springer, Berlin, 1999).
